# Beyond Post-tuberculosis Sequelae: Uncovering Common Variable Immunodeficiency in an Adult

**DOI:** 10.7759/cureus.89559

**Published:** 2025-08-07

**Authors:** Inbarasan Asaithambi, Dhanasekar Thangaswamy

**Affiliations:** 1 Pulmonology, Sri Ramachandra Institute of Higher Education and Research, Chennai, IND

**Keywords:** bronchiectasis, common variable immunodeficiency, immunodeficiency, ivig, post tuberculosis sequelae, recurrent infections

## Abstract

Common variable immunodeficiency (CVID) is a rare immunodeficiency syndrome presenting with wide manifestations and leading to a delayed diagnosis. A 40-year-old male, a case of old treated tuberculosis, presented with a productive cough and hemoptysis. He had a history of recurrent respiratory symptoms previously attributed to post-tuberculosis sequelae with bilateral bronchiectasis, which can also occur as a manifestation of underlying common variable immunodeficiency (CVID). After a detailed evaluation, serum immunoglobulin levels were markedly reduced, confirming CVID. The patient was started on intravenous immunoglobulin (IVIG) therapy every month. After a six-month follow-up, the patient was symptomatically better and had reduced hospitalizations.

## Introduction

Common variable immunodeficiency (CVID) is a type of primary immune system disorder characterized by defective B-cell differentiation, leading to a reduced production of immunoglobulins [1,2]. The clinical presentation is highly variable, often involving recurrent infections affecting the respiratory, gastrointestinal, or urinary tracts, alongside a predisposition to autoimmune disorders and malignancies [3,4]. Due to its varied manifestations, timely diagnosis is frequently delayed or missed. We present the case of a 40-year-old male ultimately diagnosed with CVID after years of recurrent infections and initial misattribution to post-tuberculosis sequelae.

## Case presentation

A 40-year-old male, civil engineer by occupation and a nonsmoker, presented to our hospital with a one-week history of cough with yellowish, non-foul-smelling, mucoid expectoration, shortness of breath (mMRC II), and low-grade intermittent fever. The patient had a history of mild hemoptysis, on and off, for three days. He also had complaints of non-bilious vomiting for two days, associated with regurgitation of food. He also had unintentional and non-quantifiable loss of weight and loss of appetite over the past few months. A general examination revealed him to be poorly built with a BMI of 16.5. Auscultation revealed bilateral coarse crepitations. Vitals were within normal limits with no signs of respiratory failure.

**Figure 1:**
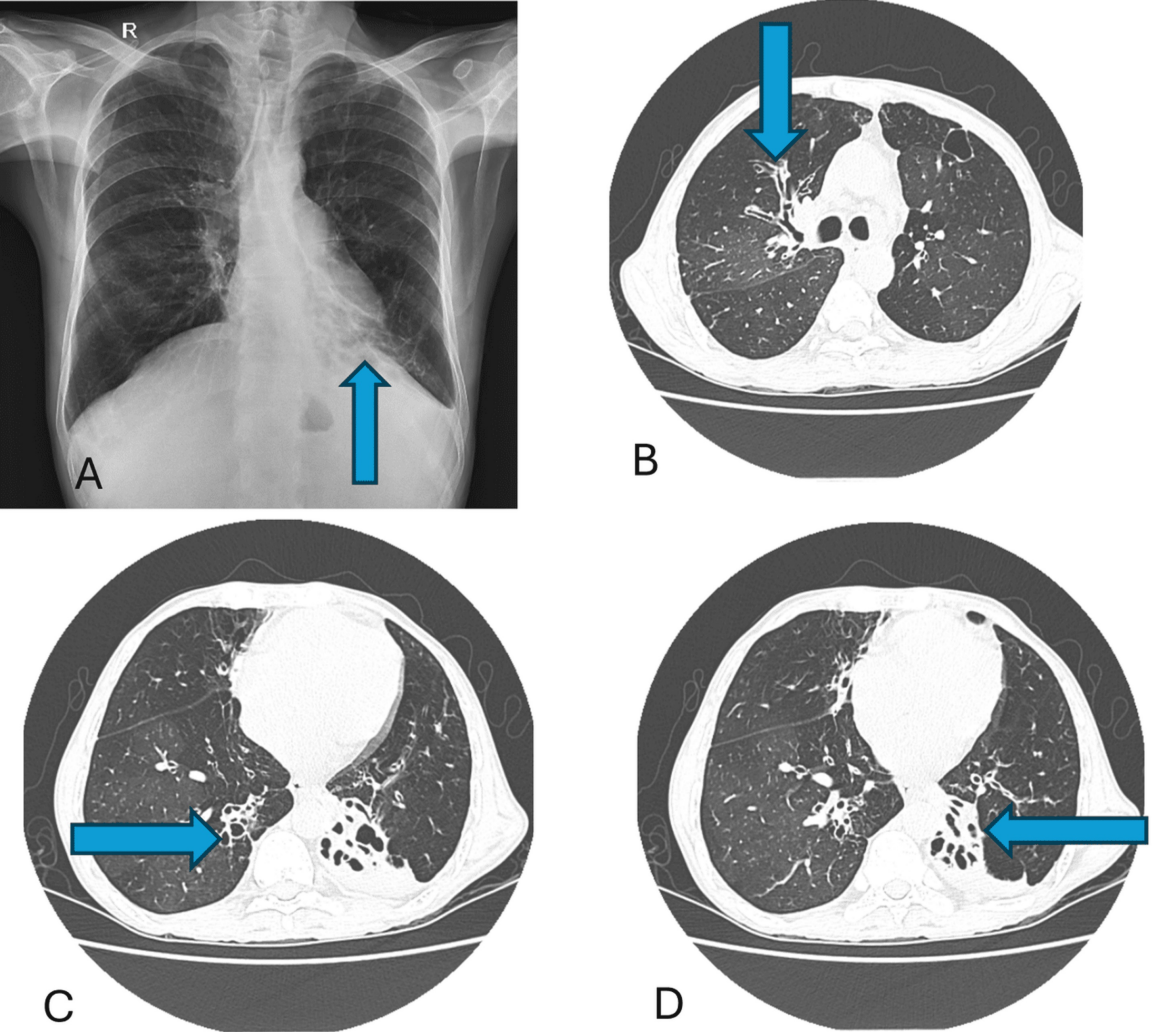
A. Chest X-ray PA view; B, C, and D. CT chest axial view Computed tomography (CT) of the chest shows bilateral bronchiectasis with mild bronchial wall thickening in the right lung and left upper lobe. It also shows left lower lobe collapse with bronchiectasis. *Blue arrows indicate bronchiectasis.*

CT pulmonary angiogram showed left lower lobe collapse, consolidation with volume loss, bronchiectasis with compensatory hyperinflation of the left upper lobe, and no evidence of primary or secondary pulmonary thromboembolism (Figure 1). Bilateral bronchiectasis and centrilobular nodules were present.

2D Echo was normal, with an ejection fraction (EF) of 67% and mild pulmonary hypertension (37 mmHg). Sputum for bacterial culture, AFB smear, and GeneXpert MTB were negative. The laboratory results are shown in Table 1. Fiberoptic bronchoscopy (FOB) was considered to rule out active endobronchial tuberculosis or other lesions. However, it was deferred because the patient’s hemoptysis was mild and self-limiting. High-resolution CT chest revealed no endobronchial pathology, and all sputum investigations for tuberculosis (acid-fast Bacilli (AFB) smear, GeneXpert Mycobacterium tuberculosis (MTB)/rifampicin (RIF), tuberculosis polymerase chain reaction (TB-PCR), and Mycobacteria growth indicator tube (MGIT) culture) were negative. FOB was planned only if hemoptysis persisted or new imaging findings emerged. Upper gastrointestinal endoscopy (UGI scopy) was performed, which showed Helicobacter (H.) pylori-associated chronic gastritis. Colonoscopy was performed in view of intermittent diarrhea and abdominal pain, which revealed the presence of rectal tubular adenoma and caecal polyp (leiomyoma). The above findings are listed in Table 2.

**Table 1:** Basic laboratory workup CBC revealed anemia and lymphopenia. LFT showed elevated ALP and GGT, reduced globulins, and an increased A: G ratio. CBC - Complete Blood Count: P - Polymorphonuclear Leukocytes - Neutrophils, L - Lymphocytes, E - Eosinophils; LFT - AST - Aspartate Aminotransferase; ALT - Alanine Aminotransferase; ALP - Alkaline Phosphatase; GGT - Gamma Glutamyl Transferase; A: G - Albumin: Globulin ratio; AFB - Acid Fast Bacilli ; MTB - Mycobacterium Tuberculosis; TB PCR - Tuberculosis Polymerase Chain Reaction; NTM - Non-tuberculous Mycobacteria; MGIT - Mycobacteria Growth Indicator Tube

Laboratory parameters	Patients value	Reference range
Complete Blood Count (CBC)		
Hemoglobin	11.9	13-17 g/dl
Total counts	8210	4000-11000/mm3
Differential count (P/L/E)	P - 75.2	45 – 70%
	L - 20.1	25 – 40%
	E - 0.1	1 – 6%
Platelets	2.26	1.5 - 4.5 lakhs/mm^3^
Renal Function Test (RFT)		
Blood Urea Nitrogen	16	6 – 20 mg/dl
Creatinine	0.7	0.7 – 1.2 mg/dl
Liver Function Test (LFT)		
Enzymes	AST – 21	< 40 IU/L
	ALT – 22	< 41 IU/L
	ALP – 255	32-120 IU/L
Total Protein	Total - 5.5	6.6 – 8.7 g/dl
	Albumin - 4.3	3.5 – 5.2 g/dl
	Globulin - 1.3	2 – 3.5 g/dl
A: G ratio	3.4	1.1 – 2.0
Total bilirubin	Total - 0.35	<1.2 mg/dl
	Direct - 0.17	<0.30
	Indirect - 0.18	0.1-1
Gamma Glutamyl Transferase	140	<60
Sputum Analysis		
AFB smear	Negative	
GeneXpert MTB	Not Detected	
Bacterial culture	No Growth	
Direct Smear Nocardia	Negative	
TB PCR (MTB+NTM)	Negative	
MGIT Culture	No Growth	

**Table 2:** Gastrointestinal symptoms workup The above test results confirmed the diagnosis of H. pylori-associated chronic gastritis, consistent with CVID-associated enteropathy. A colonoscopy was done to rule out any lymphoproliferative lesions or malignancies. UGI scopy - Upper Gastrointestinal Endoscopy; H. pylori - Helicobacter pylori; CVID - Common Variable Immunodeficiency

USG abdomen	Splenomegaly with perisplenic varices
UGI scopy	H. pylori-associated chronic gastritis
Colonoscopy	Caecal polyp – leiomyoma
	Rectal tubular adenoma

**Figure 2:**
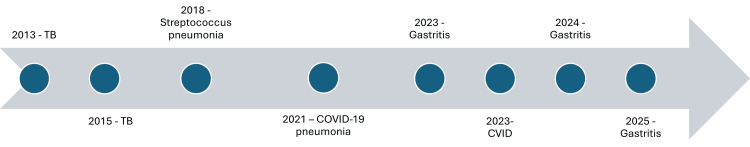
Timeline of illnesses in our patient The figure describes the timeline of illnesses in our patient. The patient had a history of recurrent respiratory and gastrointestinal infections. TB - Tuberculosis; COVID-19 - Coronavirus Disease 2019; CVID - Common Variable Immunodeficiency

After a detailed medical history and physical examination, some key findings were identified. Multiple histories of pulmonary tuberculosis in the past 10 years (twice), both previous episodes of tuberculosis were microbiologically confirmed as drug-sensitive TB, with negative rifampicin resistance on GeneXpert MTB/RIF and treatment completed with first-line ATT. During the current admission, the patient was re-evaluated for possible MDR or XDR TB given his history of recurrence. However, AFB smear, GeneXpert MTB/RIF, TB-PCR (MTB+NTM), and MGIT culture were all negative, effectively ruling out active TB or drug resistance. The patient had a history of mild COVID-19 infection, managed conservatively. History of multiple Helicobacter pylori-associated chronic gastritis in the last five years, which were treated with antibiotics and proton pump inhibitors (Figure 2).

The onset of his medical issues was 10 years earlier when he developed a chronic productive cough. Owing to the history of tuberculosis in the past, his recurrent respiratory complaints were attributed to post-tuberculosis sequelae - Bronchiectasis, and he was treated with antibiotics and oral and nebulised bronchodilators.

Over the years, despite adequate treatment, the patient presented with recurrent respiratory and gastrointestinal symptoms, and we suspected an immunodeficiency disorder. HIV was ruled out. The immunoglobulin assay revealed decreased levels of IgG, IgM, and IgA (Table 3). A diagnosis of CVID was made based on the ICON diagnostic criteria, which include reduced serum IgG with either reduced IgA or IgM, age above four years, and the presence of characteristic clinical features. Secondary causes, such as HIV, were ruled out. Although vaccine response testing was not done due to the urgency of initiating treatment, the remaining criteria were fulfilled.

**Table 3:** Immunodeficiency workup The table shows decreased levels of immunoglobulins, suggestive of CVID. IgA - Immunoglobulin A;, IgM - Immunoglobulin M; IgG - Immunoglobulin G; HIV - Human immunodeficiency virus; EBV - Epstein-Barr virus; CVID: Common variable immunodeficiency #Targeted gene sequencing: Selective capture and sequencing of the protein-coding regions of the genome/genes is performed. Mutations identified in the exonic regions are generally actionable compared to variations that occur in non-coding regions. Targeted sequencing represents an approach to detect variants present in multiple/large genes in an individual. A targeted NGS primary immunodeficiency gene panel covers antibody deficiency, combined immunodeficiency, phagocytic disorders, and complement defects. No pathogenic variants were identified; a heterozygous IKBKB variant of uncertain significance was reported. *A heterozygous missense variation in exon 11 of the IKBKB gene was detected (variants of uncertain significance).

Laboratory test	Result	Range
IgG	<7.66	700–1600 (mg/dL)
IgA	<26.9	70–400 (mg/dL)
IgM	<19.2	40–230 (mg/dL)
HIV antibody	Negative	
EBV antibody testing	Negative	
Direct and indirect Coombs tests	Negative	
Antinuclear antibody (ANA)	Negative	
Bone marrow aspirate	Hypocellular	
Primary immunodeficiency genes panel ^#^	Negative (*IKBKB gene detected)	

A targeted NGS panel for primary immunodeficiency disorders was performed. This panel screens for major monogenic immunodeficiencies, including predominantly antibody deficiencies (e.g., TNFRSF13B/TACI, ICOS, CD19, IKBKB), combined immunodeficiencies, phagocytic and innate immunity defects, and complement pathway deficiencies.

No pathogenic or likely pathogenic variants were detected. A heterozygous IKBKB variant of uncertain significance was identified.

Putting together the above findings, a diagnosis of CVID was made. The patient was treated with IV immunoglobulin 20 g (400 mg/kg) every four weeks. The patient is now symptomatically better, with a reduced number of hospital admissions per year. 

## Discussion

Definition and phenotype

CVID is one of the most prevalent symptomatic primary immunodeficiencies in adults and exhibits wide clinical and immunological heterogeneity [1,3]. It is primarily defined by decreased levels of immunoglobulin G (IgG) and either IgA or IgM [2], leading to recurrent infections and immune dysregulation. The term "variable" underscores the diversity in clinical outcomes and immunologic profiles, which can range from asymptomatic individuals to those with severe organ involvement and complications [3].

The age of onset of symptoms occurs between the second and third decades of life [5]. As age increases, the prevalence of complications also increases substantially, and it is necessary to differentiate those complications due to previous infections from complications due to underlying immune dysregulation [3]. Hence, complications are classified as immune dysfunction-related consequences and structural damage due to prior infections.

Respiratory infections and bronchiectasis

Individuals with CVID are particularly vulnerable to infections by encapsulated bacteria, such as Haemophilus influenzae and Streptococcus pneumoniae, often resulting in recurrent sinusitis and pneumonia [2]. Over time, repeated pulmonary infections can cause structural lung damage, including bronchiectasis [6]. Bronchiectasis in CVID patients is thought to occur due to two reasons: recurrent lower respiratory tract infections or a prior history of serious infections, like pneumonia and septicemia, in the past [6]. Bronchiectasis in CVID reflects chronic airway injury from recurrent infections, and in our patient, likely represents a combination of post-tuberculosis and CVID-related damage.

Our patient's presentation with recurrent respiratory infections and bronchiectasis raised suspicion for an underlying immunodeficiency, which was confirmed by low immunoglobulin levels consistent with CVID. At the same time, low levels of immunoglobulins are not the sole predictor of this outcome. The development of bronchiectasis could be attributed to either underlying immunodeficiency or as a sequela to prior severe infections such as tuberculosis.

Gastrointestinal manifestations

About 15% or more of patients with CVID can present with a variety of gastrointestinal symptoms, such as altered bowel movement, recurrent GI infections, like Giardiasis or Campylobacter [7], autoimmune conditions, and malignancies. In our case, the patient had frequent episodes of vomiting. UGI scopy revealed chronic H. pylori-associated gastritis, which required treatment with antibiotics and proton pump inhibitors. The patient also had frequent episodes of loose stools. Colonoscopy revealed a cecal polyp. Biopsy and immunohistochemistry showed features suggestive of leiomyoma. Gastrointestinal manifestations are an important contributor to the non-malignant morbidity associated with CVID [2,8].

Autoimmune and lymphoproliferative complications

CVID can be associated with a wide range of immune dysregulation syndromes, including autoimmune cytopenias (immune thrombocytopenic purpura (ITP), autoimmune hemolytic anemia (AIHA)), thyroiditis, and systemic inflammatory conditions such as granulomatous disease [6]. An infection triggers an inflammatory response that fails to be downregulated once the infection is resolved. Persistent lymphadenopathy or hepatosplenomegaly may reflect underlying lymphoproliferation or immune activation rather than malignancy [2], although both must be ruled out through appropriate workup.

Malignancy risk

There is an elevated risk of malignancy in CVID, particularly non-Hodgkin lymphomas, which tend to occur in middle-aged to older adults [6]. The increased risk is believed to result from chronic immune activation, impaired immunosurveillance, and underlying lymphoproliferative processes. This risk necessitates regular monitoring for early signs of hematologic or solid organ malignancies [9].

Diagnosis

The most recent International Consensus Document (ICON) guidelines list five criteria for CVID diagnosis [10]: (1) IgG level less than two standard deviations for two measurements more than three weeks apart; (2) either low IgA or low IgM, (3) poor antibody responses to vaccination, (4) patient age above four years, (5) no secondary causes of hypogammaglobulinemia.

In contrast, the European Society for Immunodeficiencies (ESID) [10] criteria emphasize the (i) presence of low IgA, (ii) usage of low switched memory B cells, instead of measurement of antibody response to vaccine, (iii) exclusion of profound T-cell deficiency, and (iv) a clinical manifestation of disease such as an increased susceptibility to infection, autoimmune manifestations, granulomatous disease, or unexplained polyclonal lymphoproliferation, or an affected family member with antibody deficiency.

Management

Management involves regular follow-ups of patients for both common and rare complications of CVID. This is done with the aim of detecting clinical phenotypes such as autoimmune cytopenia, lymphoproliferative disorders, or enteropathy at the earliest, as they have reduced survival [6]. Once infections are identified, they should be treated with appropriate antibiotics. Nutritional and vitamin supplementation should be given for those with gastrointestinal involvement, and disease-specific treatment for autoimmune or malignant complications. Conditions such as bronchiectasis warrant the use of prophylactic long-term antibiotics [11].

Standard therapy for CVIDs is the supplementation of immunoglobulin IgG via intravenous or subcutaneous routes [3]. The usual dosage is 0.4 to 0.6 g/kg of immunoglobulin per month, in divided doses [3]. The dose is titrated based on clinical response and trough IgG levels. Routine follow-up is essential to identify evolving complications, particularly those that may impact long-term prognosis.

Management of complications

Not all complications require therapy. The need for additional therapy depends on a risk assessment for each patient. Malabsorption is one of the debilitating and life-threatening complications of enteropathy [3]. Patients must be routinely screened and treated for nutrition, including vitamin deficiencies. Other complications, such as infections, autoimmune pathologies, and malignancies, are managed according to disease-specific management guidelines.

## Conclusions

The heterogeneous clinical presentation of CVID often leads to delays in diagnosis, with patients frequently being treated for recurrent infections or their complications without recognition of the underlying immunodeficiency. In this case, the patient was repeatedly managed for presumed post-tuberculosis sequelae before CVID was identified as the root cause. This highlights the critical importance of maintaining a high index of suspicion for primary immunodeficiency disorders in adults presenting with recurrent infections and bronchiectasis, especially when conventional treatments fail to yield lasting improvement. Early diagnosis and appropriate immunoglobulin replacement therapy can significantly reduce morbidity and improve quality of life. Addressing the current gaps in diagnostic awareness and clinical evaluation strategies is essential for the timely recognition and effective management of CVID.

## References

[1] Bonilla FA, Geha RS (2009). Common variable immunodeficiency. Pediatr Res.

[2] Pescador Ruschel MA, Vaqar S (2025). Common variable immunodeficiency. StatPearls [Internet].

[3] Chapel H, Cunningham-Rundles C (2009). Update in understanding common variable immunodeficiency disorders (CVIDs) and the management of patients with these conditions. Br J Haematol.

[4] Cunningham-Rundles C (2008). Autoimmune manifestations in common variable immunodeficiency. J Clin Immunol.

[5] Tam JS, Routes JM (2013). Common variable immunodeficiency. Am J Rhinol Allergy.

[6] Chapel H, Lucas M, Lee M (2008). Common variable immunodeficiency disorders: division into distinct clinical phenotypes. Blood.

[7] Cunningham-Rundles C (2010). How I treat common variable immune deficiency. Blood.

[8] Daniels JA, Lederman HM, Maitra A, Montgomery EA (2007). Gastrointestinal tract pathology in patients with common variable immunodeficiency (CVID). A clinicopathologic study and review. Am J Surg Pathol.

[9] Cabañero-Navalon MD, Garcia-Bustos V, Balastegui-Martin H (2024). The impact of immune dysregulation on the risk of malignancy in common variable immunodeficiency: insights from a multicenter study. Front Immunol.

[10] Lee TK, Gereige JD, Maglione PJ (2021). State-of-the-art diagnostic evaluation of common variable immunodeficiency. Ann Allergy Asthma Immunol.

[11] Choi H, McShane PJ, Aliberti S, Chalmers JD (2024). Bronchiectasis management in adults: state of the art and future directions. Eur Respir J.

